# Actomyosin Is the Main Driver of Interkinetic Nuclear Migration in the Retina

**DOI:** 10.1016/j.cell.2009.06.032

**Published:** 2009-09-18

**Authors:** Caren Norden, Stephen Young, Brian A. Link, William A. Harris

**Affiliations:** 1 Department of Physiology, Development and Neuroscience, Cambridge University, Downing Street, Cambridge CB2 3DY, UK; 2 Department of Cell Biology, Neurobiology, and Anatomy, Medical College of Wisconsin, 8701 Watertown Plank Road, Milwaukee, WI 53226, USA

**Keywords:** MOLNEURO, CELLBIO

## Abstract

Progenitor cell nuclei in the rapidly expanding epithelium of the embryonic vertebrate central nervous system undergo a process called interkinetic nuclear migration (IKNM). Movements of IKNM are generally believed to involve smooth migration of nuclei from apical to basal and back during the G1 and G2 phases of the cell cycle, respectively. Yet, this has not been formally demonstrated, nor have the molecular mechanisms that drive IKNM been identified. Using time-lapse confocal microscopy to observe nuclear movements in zebrafish retinal neuroepithelial cells, we show that, except for brief apical nuclear translocations preceding mitosis, IKNM is stochastic rather than smooth and directed. We also show that IKNM is driven largely by actomyosin-dependent forces as it still occurs when the microtubule cytoskeleton is compromised but is blocked when MyosinII activity is inhibited.

## Introduction

An epithelium is a sheet of polarized cells that extend from an apical surface to a basal lamina. Mitotic events in a proliferating epithelium are restricted to the apical pole, where the centrosomes are located. A hallmark of the vertebrate neuroepithelium is interkinetic nuclear migration (IKNM), originally described by Sauer (1935). Sauer suggested a model in which mitosis at the apical surface is followed by a smooth basal descent of the nucleus during G1, and, after S phase, a gradual ascent back to the apical surface for the next mitosis, like an elevator descending to the bottom floor, pausing, and then smoothly rising again (Miyata, 2008).

The dramatic elongation of progenitor cells and the variation in the apicobasal position of their nuclei gives the vertebrate neuroepithelium a pseudostratified (multilayered) appearance. It is suggested that this cellular morphology has arisen to maximize the density of generative cells per unit area of apical surface (Fish et al., 2008). In line with this idea, one function of IKNM may simply be to the move nuclei of postmitototic progenitors away from the apical surface, making room for other mitotic cells. Additionally, it has recently been reported that IKNM influences neurogenerative cell divisions through Notch signaling, which is polarized toward the apical surface of the epithelium (Del Bene et al., 2008; Murciano et al., 2002).

If Sauer's model of IKNM is correct, S phase nuclei should be restricted to basal positions in the neuroepithelium, yet S phase nuclei occupy a range of positions along the apicobasal axis of the epithelium (Baye and Link, 2007). Moreover, the same report shows that the distances that nuclei migrate basally are highly variable. These results raise questions about how the movements of IKNM correlate with the particular phases of the cell cycle, and whether the movements are really monotonic, as Sauer proposed.

The force-generating mechanisms that move the nucleus during IKNM are largely unknown. Microtubules (MTs) in yeast move nuclei via pulling or pushing mechanisms (Kusch et al., 2003; Tran et al., 2001). Pulling usually requires centrosome-microtubule collaboration in proximity to the nucleus. In neuroepithelial cells, however, centrosomes remain at the apical pole of the cell, distant from the nucleus, during the majority of the cell cycle (Chenn et al., 1998). Tubulin polymerization itself produces a pushing force, but in vitro studies indicate that MTs exceeding 10 μm buckle under compression (Dogterom et al., 2005). MTs would need to resist buckling at more than twice this length in order to push nuclei the distances observed in zebrafish retinal neuroepthelial cells.

Perhaps IKNM is powered by motor proteins along a MT scaffold as proposed by Xie et al. (Xie et al., 2007). Indeed, two recent publications implicate dynein/dynactin, a ubiquitous minus end-directed MT-associated motor protein complex, in apical migration during IKNM (Del Bene et al., 2008; Tsai et al., 2005). The first shows that downregulating Lis1 (a dynein interactor) interferes with IKNM (Tsai et al., 2005), while the second shows that in dynactin1 mutant zebrafish (*mok*), nuclei reach more basal positions than wild-type embryo nuclei (Del Bene et al., 2008). Neither study rules out the possibility that other cytoskeletal components also influence IKNM.

Actomyosin networks generate force in many different cellular contexts, so it is reasonable to suspect that they might also be involved in IKNM. Indeed, Webster and Langman (Webster and Langman, 1978) found defects in nuclear positioning, such as nuclei that are too far basal, after exposure of chick neuroepithelia to actin depolymerising drugs.

Taking advantage of the transparency of zebrafish embryos by using live imaging approaches, we carried out a methodical investigation of IKNM movements and the molecular mechanisms that are involved in driving them. We focused on the zebrafish retina, a lateral extension of the CNS, because of its particular imaging accessibility. We show that except for brief periods near mitosis, IKNM movements are stochastic. Mechanistically, we find that MTs and their motor proteins play a minor role in IKNM but that most nuclear movement is generated by actomyosin activity.

## Results

### IKNM Is a Largely Stochastic Process

**Figure 1:**
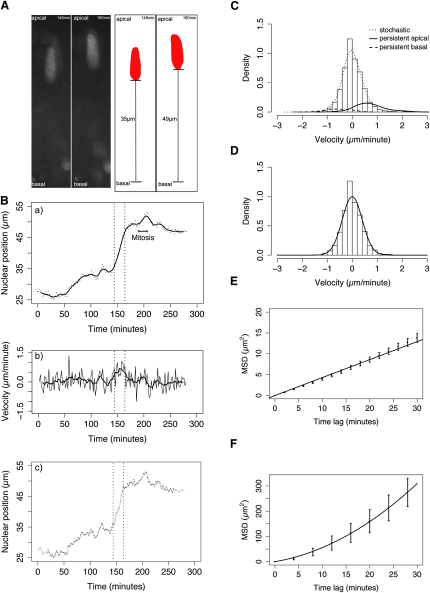
Statistical Analysis Reveals Stochastic and Directed Components of IKNM (A) Schematic illustrating measurement of nuclear position (distance from the basal lamina). Raw data and corresponding measurements (black lines) are shown for two example time points. (B) Trajectory (dots) and trend (moving average, line) of a single nucleus (shown in A) exhibiting distinct classes of motion are shown (a), corresponding instantaneous velocity series (thin line) with moving average (thick line) (b), and automated identification of transient periods of rapid persistent movement (red) in a background of stochastic movement (black) (c). (C) Velocity distribution of pooled nuclear movements in control embryos (curves show nonparametric density estimates scaled in proportion to the number of nuclear displacements representing the motion type, persistent motion curves are enlarged by a factor of ten). (D) Stochastic motion velocity distribution for pooled nuclear movement in control embryos with a normal fit. (E) Stochastic motion mean squared displacement (MSD) profile (error bars show 95% confidence interval) with linear fit for pooled control nuclei. (F) Premitotic persistent apical motion MSD profile (error bars show 95% confidence interval) with parabolic fit for pooled control nuclei.

We began this study by testing the model reported in numerous reports and text books in which, after mitosis at the apical surface, nuclei migrate basally during G1 in a smooth monotonic fashion, pause in S phase, and monotonically return to the apical surface during G2 (Miyata, 2008; Murciano et al., 2002; Nowakowski, 2005; Sauer, 1935). Starting at 28–32 hours postfertilization (hpf), we tracked the location of nuclei, marked by H2B-RFP in the developing zebrafish retina (Figures 1A and 1Ba). Nuclear locations were sampled at 2 or 5 min intervals at different phases of the cell cycle. Visual inspection suggested that these trajectories contained at least two distinct types of motion (Figure 1B). The first type is persistent and rapid; the orientation of successive steps is positively correlated and the step velocities correspond to values in the tails of the measured velocity distributions (within dotted lines, and Figure 1C). The second type of motion is stochastic; the direction of movement changes frequently, apparently at random intervals (outside dotted lines, and Figures 1C and 1D).

We employed an automated approach based on a moving analysis window (Huet et al., 2006) to identify periods of rapid persistent motion within individual trajectories. We incorporated a threshold based on simulated one-dimensional random walk analyses to control for false positives (Saxton and Jacobson, 1997). In control embryos, persistent rapid nuclear movements occur transiently in both apical and basal directions. Persistent basal movements are observed with low frequency and last for about 7 min (7 min ± 2 min, mean ± SD). They tend to occur soon after cell division close to the apical pole and transport nuclei an average distance of 18 ± 8 μm, approximately 40% of the epithelium. Persistent apical movements occur in G2 prior to cell division, last for 5 to 30 min (14 min ± 8 min, mean ± SD) and transport nuclei a distance of 30% to 70% of the epithelium. They initiate from a range of positions along the apicobasal axis but consistently transport nuclei to the apical surface (Figure 1Bc). Previous estimates indicate that the cell cycle for zebrafish retinal progenitor cells at this developmental stage ranges between 4 and 11 hr (Baye and Link, 2007). This means that the nuclei of these cells spend less than 10% of the cell cycle undergoing persistent directed movement as defined by our algorithm. During the other 90% of the cell cycle, nuclear motion is stochastic.

Restricting our analyses to phases of stochastic movement, we find that the distribution of nuclear velocities is well described by a normal distribution with mean ≈ 0 (Figure 1D) and that the moving average of the instantaneous velocity series is stationary (≈0 at all times) (Figure 1Bb). Furthermore, the mean squared displacement (MSD) of nuclei (Figure 1E) has a linear relationship with elapsed time, diagnostic of a particle undergoing random motion. This is in contrast to the MSD profile calculated for periods of persistent motion prior to mitosis (Figure 1F), which shows a positive curvature indicative of directed motion. Thus, by these statistical criteria, it appears that IKNM is a stochastic process, similar to a random walk or Brownian motion, punctuated with transient directed movements surrounding cell division.

### Stable Polarized MTs Span the Retinal Epithelium and Enclose the Nucleus

**Figure 2:**
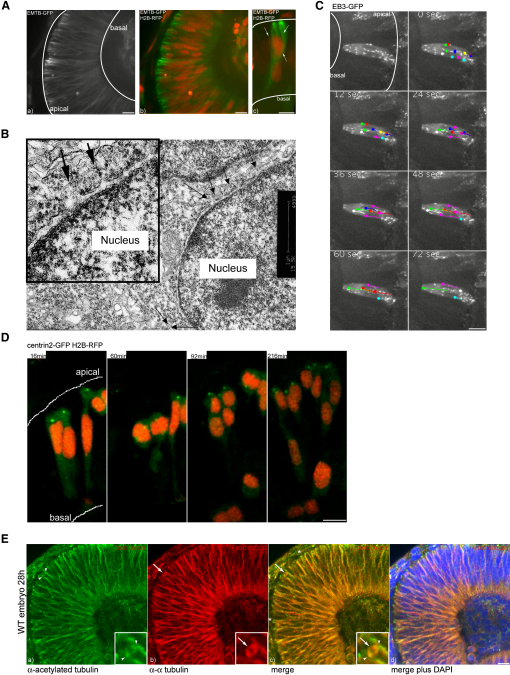
An Oriented Microtubule Cage Surrounds the Nuclei of Retinal Neuroepthelial Cells (A) MTs labeled with EMTB-GFP span the whole epithelium (a) and surround nuclei labeled by H2B RFP (b). A Three-dimensional reconstruction of MTs that envelope a nucleus is shown (c). (B) Ultrastructure of MTs in interphase close to the nuclear membrane. MTs are labeled by arrows. (C) EB3-GFP as a marker for polymerizing MT tips. Dots at the end of lines mark the last position of EB3-comets followed over time. (D) Centrin dots marked by Centrin2-GFP stay in apical positions throughout the movie. H2B-RFP marks nuclei. (E) Antibody staining of stable (acetylated) Tubulin (green) (a) versus α-tubulin (red) (b). A merge of (a) and (b) is shown in (c). DAPI (blue) counterstaining shows epithelial morphology (d). Arrows label dynamic spindle structures, arrowheads label stable spindle pole centrosomes, and asterisks label stable basal bodies. Scale bars represent 10 μm.

We began to investigate the mechanisms responsible for IKNM by examining the microtubule cytoskeleton. A GFP-tagged version of the microtubule binding protein E-MAP-115 (ensconsin), EMTB-GFP (Bulinski et al., 1999), was used to label filamentous tubulin. EMTB-GFP revealed that MTs span the entire apicobasal axis of the epithelium (Figure 2A). When cells were viewed at 40 s intervals in confocal projections, no obvious temporal changes in EMTB-GFP distribution were observed, indicating the existence of a stable MT population (Figure 2A and Movie S2 available online). Three-dimensional reconstructions show nuclei surrounded by MTs (Figure 2A). Transmission electron micrographs of zebrafish retinal sections from comparable stages show MTs within 100 nm of the nuclear membrane (Figure 2B).

To examine the polarity of these MTs, we imaged the plus end marker EB3 fused to GFP (Stepanova et al., 2003). Confocal time-lapse movies show that polymerization of MTs is directed away from the centrosome (Figure 2C), which is anchored at the apical pole of these cells throughout interphase. At metaphase, when the nucleus has migrated to the apical surface of the epithelium, the centrosomes localize to the spindle poles at the metaphase plate (centrosomes are labeled with centrin2-GFP). As soon as the two cells separate, a single centrosome in each daughter cell returns to the apical pole (Figure 2D and Movie S4). Since MTs grow from apical to basal (Figure 2C and Movie S3), it follows that MTs in interphase are polarized within the epithelium such that their minus ends point apically and their plus ends point basally.

The number of basally directed EB3-GFP “comets” suggests a significant pool of dynamic MTs, while our EMTB observations suggest that there is also a pool of stable MTs. We therefore assayed the relative abundance and localization of the different subpopulations. Antibodies to acetylated tubulin decorate stable MTs (Takemura et al., 1992), while antibodies to α-tubulin mark all MTs as well as cytosolic tubulin monomers. That acetylated tubulin was not associated with dynamic MTs is demonstrated by the fact that antibodies to acetylated tubulin mark the stable spindle pole bodies but not the dynamic spindle structures that link these to the metaphase plate, the latter being labeled only by antibodies to α-tubulin (arrows and inset in Figure 2E). Comparison of the two markers in interphase suggests that most of the MTs in the zebrafish retinal neuroepithelium are acetylated and stabilized.

### The Major Features of IKNM Are Observed in the Absence of Dynactin Function

The stable and oriented population of MTs enclosing the nucleus could, in principle, allow nuclear transport in both basal and apical directions, provided that appropriate plus end- and minus end-directed motors link the nucleus to neighboring MTs. Indeed, a recent report demonstrates that in the zebrafish *mok* (dynactin-1) zygotic mutant, individual nuclei translocate to more basal positions during the course of their migration relative to nuclei in wild-type embryos (Del Bene et al., 2008), thus implicating the dynein/dynactin complex in IKNM. To understand the dynamics of this phenotype in more detail, we blocked the function of the dynein/dynactin complex cell autonomously in a temporally controlled way with a GFP-tagged dominant-negative (DN) version of the human dynactin-1 subunit p150/Glued (Vaughan et al., 2001) under the control of the zebrafish hsp70 promoter. This human p150 is highly similar to the zebrafish protein and as expected, labels mitotic spindles in zebrafish (Figures S1A and S1B).

**Figure 3:**
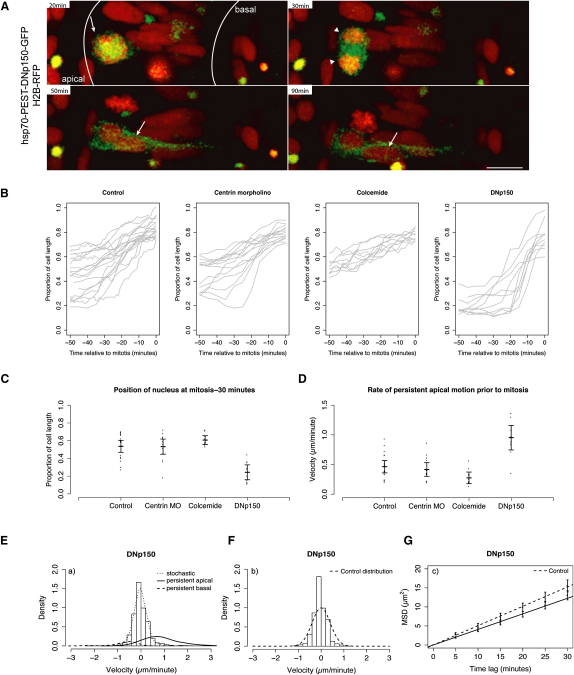
Stochastic and Directed IKNM Movements Occur in Dynein/Dynactin-Compromised Neuroepithelial Cells (A) H2B-RFP marked nuclei of hsp70-PEST-DNp150-GFP-expressing cells migrate to the apical pole (arrows), exhibit successful mitosis and cytokinesis (arrowheads), and then migrate basally (arrows). (B) Raw nuclear trajectories showing nuclear migration during the 50 min preceding formation of a metaphase plate. (C) Nuclear positions 30 min before formation of a metaphase plate. Each dot represents one nucleus. Error bars show the mean and 95% confidence intervals. (D) Premitotic (−30 to 0 min before formation of metaphase plate) velocity estimates. Each dot represents velocity of one nucleus. Error bars show the mean and 95% confidence intervals. (E) Velocity distribution of nuclear movements in DNp150-expressing cells (curves show nonparametric density estimates scaled in proportion to the number of nuclear displacements representing the motion type; persistent motion curves are enlarged by a factor of ten). (F) Stochastic motion velocity distribution for nuclear movement in DNp150-expressing cells. (G) Stochastic motion MSD profile with linear fit (error bars show 95% confidence intervals) for nuclear movement in DNp150-expressing cells. Scale bars represent 10 μm.

When embryos are heat shocked at 24 hpf, expression of DNp150 becomes detectable 2 hr later in a subset of neuroepithelial cells. When we measured the positions of nuclei of DNp150-expressing cells 30 min before formation of the metaphase plate, we found that these nuclei occupy a more basal position than the nuclei of control cells, phenocopying the *mok* mutant (Figure 3C). All mitoses in these DNp150-expressing cells, however, were seen to occur at the apical surface of the neuroepithelium, just as in control cells (Figure 3A and 3B). We therefore tracked nuclei for 50 min before the formation of the metaphase plate, and we found that nuclei of DNp150-expressing cells, similar to control cells, move apically and undergo mitosis at apical locations (Figure 3B).

The velocity histogram and the slope of the MSD profile of stochastic periods of IKNM in DNp150-expressing cells proved to be similar to that seen in control cells (Figures 3E–3G). We did, however, observe some episodes of very rapid basally directed motion in DNp150-expressing cells (Figure 3E). This suggests that dynein/dynactin normally counteracts such movements in control cells, preventing the nuclei of neuroepithelial cells from undergoing sudden translocations to basal positions during interphase.

These data argue that rapid persistent apical nuclear migration preceding mitosis is not dependent on dynein/dynactin function (Figures 3A–3D). In fact, the rate of persistent apical nuclear motion prior to division is higher in DNp150-expressing cells (Figures 3C and 3D). Also, the stochastic movement that comprises the majority of interphase IKNM (Figures 3E–3G) occurs independently of dynein/dynactin function. The slight decrease in MSD compared to the control situation can probably be explained by the fact that nuclei in DNp150-expressing cells spend most of their cell cycle at very basal positions, where they have less freedom of stochastic movement than control cells have. Our results confirm that dynein-dependent motors thus play a role in IKNM; however, it is clear that this role is dispensable for either rapid apical or stochastic movements that constitute the majority of nuclear movements in IKNM.

### IKNM in the Absence of Stable MTs

Because dynein/dynactin is the main MT associated minus end-directed motor in cells, yet all major dynamic features of IKNM are retained in DNp150-expressing cells, we decided to extend our investigation of whether MTs and their associated motors are necessary for IKNM. We first knocked down the centrosomal protein, centrin2, which forms an interface between MTs and the centrosome (Bornens, 2002), using translation and splice blocking morpholinos directed against centrin2. RT-PCR shows that the splice-disrupting morpholinos nearly completely downregulated the normal centrin message (Figure S2A). The gross phenotypes induced by either translation-disrupting or splice-disrupting morpholinos are similar. At 24 hpf, morphants display a slightly thinner retinal neuroepithelium (data not shown).

**Figure 4:**
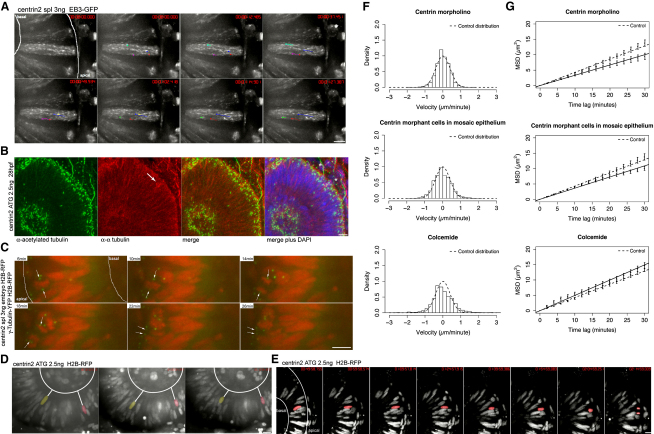
IKNM Is Relatively Normal in the Absence of Stable Microtubules (A) Polymerizing MT tips are stained by EB3. Centrin2 morphants show the same EB3-GFP kinetics as control embryos in Figure 1B. Dots at the end of lines mark the last position of EB3 comets followed. (B) Antibody staining of α-tubulin (red) and acetylated tubulin (green) as in Figure 2C. Acetylated tubulin stains basal bodies (asterisks), but no stabilized MTs are observed spanning the epithelium (a). α-tubulin antibodies label mitotic spindles (arrow) and residual dynamic tubulin (b). A merge of (a) and (b) is shown in (c). Asterisks label stable basal bodies that are only stained by acetylated and not α-tubulin. DAPI (blue) counterstaining shows epithelial morphology (d). (C) Images of Movie S6. Centrin2 morphants perform mitosis and cytokinesis at the apical membrane of the epithelium, after mitosis nuclei marked by H2B-RFP move away from this side. Centrosomes marked by γ-tubulin first serve as spindle pole bodies and then move back to apical side of epithelium (arrows). (D) Images of Movies S7 and S8. Nuclei labeled by H2B-RFP move in apical and basal direction in centrin-2 morphants, indicated by white lines between false color red and yellow nuclei and the basal membrane of the epithelium. (E) Nuclei still undergo rapid apical migration before mitosis as seen in the control cells. (F) Stochastic motion velocity distributions for centrin morphant embryos, mosaic centrin morphant cells in control epithelium, and Colcemide-treated embryos. (G) Stochastic motion MSD profile with linear fit (error bars show 95% confidence intervals) for centrin morphant embryos, mosaic centrin morphant cells in control epithelium, and Colcemide-treated embryos. Scale bars represent 10 μm.

In centrin2 morphants, centrosomes still localize to the apical surface (Figures 4A and 4C) and MTs polymerize in an apical to basal fashion at the same rate as in control embryos (Figure 4A and Movie S5). However, staining of stable MTs with anti-acetylated tubulin shows a striking difference between centrin2 morphants and the control embryos. While in control cells, acetylated MTs span the entire length of the cell (Figure 2E), centrin2 morphant cells only show remnants of these structures (Figure 4B). Immunoreactivity in basal bodies serves as an internal control for these experiments. α-tubulin immunoreactivity still labels spindle structures (arrow in Figure 4B), interphase dynamic MTs, and nonpolymerized tubulin in centrin2 morphant cells. These results show that although MT polymerization occurs in centrin2 morphants, the formation of a stable cage of MTs surrounding the nucleus is prevented.

The dynamics of IKNM in the absence of stable MTs were imaged with γ-tubulin-YFP (centrosomes) and H2B-RFP (nucleus). As the translational morphants in general showed a stronger phenotype than the splice morphants, we concentrated our analysis on this construct and refer to it as centrin morphant in graphs. (Data for the splice morphants can be found in Figure S2B.) We first noted that mitoses still happen at the apical side (Figure 4C and Movie S6) and that fast apical migration before mitosis still occurs in centrin morphant embryos (Figures 4E, 3B, and 3D and Movie S8), with basal positions 30 min before mitosis being remarkably similar in control and centrin2 morphants (Figure 3F).

The analysis of trajectories of nuclear movements was used to compare the stochastic movements of IKNM in centrin morphants to those in control embryos (Movie S7). The slope of the MSD in the centrin2 ATG morpholino is slightly less steep than that seen in control embryos (Figure 4G). To explore whether this effect was due to the slightly thinner epithelium in centrin morphants, we analyzed the consequences of centrin2 knockdown in mosaic epithelia. We injected the centrin2 morpholino into a subset of cells that also express H2B-RFP. Nonmorphant cells express H2B-YFP. Thus nuclear motion can be followed for both conditions within the same embryos. Antibody staining shows that control cells have stabilized MT cytoskeletons whereas morphant cells do not (Figure S2C). Velocity distributions and MSDs are comparable between full morphant and mosaic conditions (Figures 4F and 4G), while data for control cells do not differ from data for control cells in nonmosaic epithelia (data not shown). This argues that the slight reduction in the MSD of centrin morphant cells is not due to the thinning of the retinal neuroepithelium. It suggests instead an alternative explanation, consistent with our dynein results, that stable MTs play a minor role in generating nuclear movements during IKNM.

### IKNM in the Complete Absence of MTs

**Figure 5:**
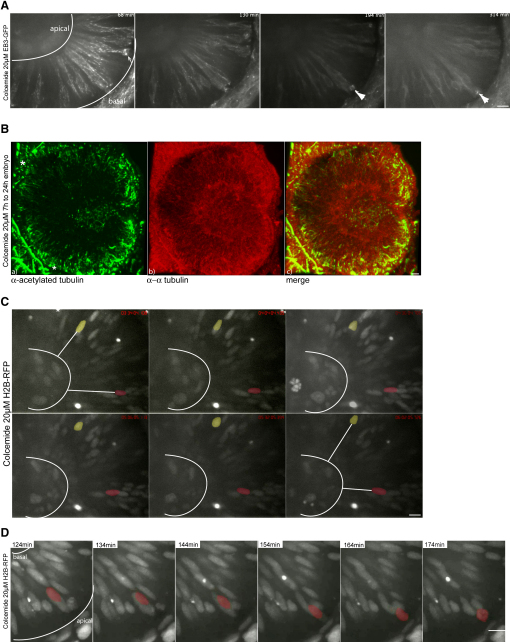
IKNM Occurs in the Complete Absence of Microtubules (A) Images of Movie S10. Polymerizing MTs are stained by EB3. Colcemide-treated (20 μM) embryos show reduced MT polymerization starting 120 min after drug addition. Shortly after drug addition, spindles are still formed and cells divide (arrow). Colcemide treatments of 120 min and longer arrest cells in metaphase for the rest of the observation time (arrowheads). (B) Antibody staining of α-tubulin (red) and acetylated tubulin (green) like in Figure 2C in embryos treated with 20 μM Colcemide. Acetylated tubulin antibodies label stable basal bodies (asterisks), but no other stable structures are seen (a). α-tubulin antibodies label cytoplasmic tubulin (b). A merge of (a) and (b) is shown in (c). Asterisks label stable basal bodies that are only stained by acetylated not by α-tubulin. (C) Images of Movie S11. H2B-RFP-labeled nuclei are able to move in apical and basal direction in epithelia treated with 20 μM Colcemide, indicated by white lines between false colored red and yellow nuclei and the basal membrane of the epithelium. (D) Images of Movie S12. Nuclei still undergo rapid apical migration before forming a metaphase plate as seen in the control cells. Scale bars represent 10 μm.

We wondered whether the IKNM seen in centrin2 morphants might depend on the remaining dynamic MTs. To explore this possibility, we depleted cells of all MTs by adding Colcemide. Colcemide is a derivate of colchicines (Goldman, 1971) and has less devastating consequences on epithelial integrity than the more commonly used compound Nocodazole. To see whether Colcemide affects MTs in the retinal neuroepithelium, we imaged EB3-GFP after bath application at 20 μM. MT polymerization is severely reduced after 120 min, and by 18 0min is no longer detectable (Figure 5A and Movie S10). Embryos treated with 20 μM Colcemide for 7 hr, the average time of our time lapse, also lack acetylated tubulin staining, indicating that stable MTs are destroyed by drug treatment (Figure 5B). We interpret the residual α-tubulin staining to be unpolymerized cytosolic tubulin.

Surprisingly, results from trajectory analysis of Colcemide-treated embryos were similar to those from control embryos. Colcemide-treated retinal neuroepithelial nuclei are still able to undergo IKNM, although they arrest in M phase at the apical side because no spindle formation occurs (Figure 5C and Movie S11). In G2, they undergo directed apical migration (Figure 5D and Movie S12). The average nuclear position 30 min prior to M phase is slightly less basal, and the average speed of apical migration is slightly slower than that in control cells. However, we did not detect significant differences in these measures (Figures 3C and 3D).

For the stochastic phases of movement, the velocity histogram of Colcemide-treated embryos is slightly broader, perhaps because of a lack of mechanical constraints of a MT cytoskeleton. We detected no significant differences between Colcemide-treated and control embryos in MSDs, (Figures 4E and 4F). Thus, in dynein-compromised cells, cells lacking stabilized MTs, and cells lacking all MTs, the core features of IKNM dymanics remain and are similar to those in control cells.

### Location of Actin and Active MyosinII Suggests a Role of Actomyosin Forces in Apically Directed IKNM

**Figure 6:**
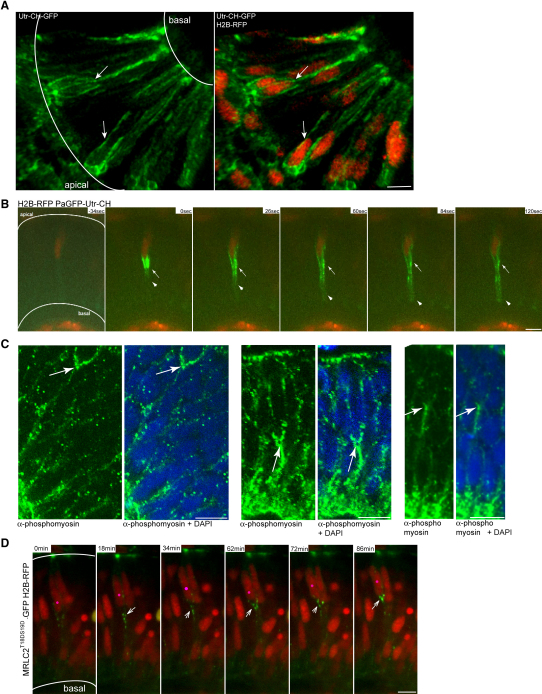
Actomyosin Is Active Basal to Apically Migrating Nuclei in the Retinal Neuroepithelium (A) Confocal image three-dimensional reconstruction of GFP-Utr-CH labeling filamentous actin and H2B-RFP labeling nuclei. GFP-Utr-CH is seen at adhesion sites and plasma membranes. Arrows label prominent actin accumulation basal to nuclei. (B) Images from Movie S14. Nuclei are labeled by H2B-RFP. After photoactivation of PaGFP-Utr-CH, it distributes more notably to the basal side of the plasma membrane beneath nucleus (arrowheads). Stable accumulations are seen basally of nucleus (arrow). (C) Antibody staining with Phospho-Myosin Light Chain 2 antibodies for activated MyosinII (green) and plus DAPI (blue). Arrows mark basal accumulations of activated MyosinII. (D) Images from Movie S15. Constitutively activated Myosin Light Chain is labeled by MRLC2^T18DS19D^-GFP. Nuclei are labeled by H2B-RFP. Pink dot labels nucleus that migrates apically. Arrows label activated Myosin Light Chain dots that move apically basal of the nucleus. Scale bars represent 10 μm.

As IKNM movements appear relatively normal in the absence of MTs, we turned our attention to the actin cytoskeleton and the associated Myosin motor proteins. Actomyosin generates force in many different nonmuscular contexts such as cytokinesis, cell migration, and apical constriction of epithelial cells during morphogenesis. To characterize the actin network in retinal neuroepithelial cells, we used a fluorescent marker based on the calponin homology domain of Utrophin (Utr-CH-GFP) (Burkel et al., 2007), which enabled us to visualize F-actin without the cytosolic background of G-actin. Confocal imaging revealed that F-actin localizes to the cortex of neuroepithelial cells. However, accumulation of actin was often observed basal to the nucleus (Figure 6A, arrows). Basal perinuclear localization persisted throughout interphase. During mitosis and cytokinesis, actin is distinctly absent from the basal process. Once cytokinesis is completed, actin rapidly relocalizes to the cortices and the basal process of both mother and daughter cells (Figure S3A and Movie S13).

To investigate actin dynamics in more detail, we used a photoactivatable GFP-Utr-CH (PaGFP-Utr-CH) (Burkel et al., 2007). This construct enables focal labeling of actin and characterization of cytosolic actin mobilization. Spot activation reveals filamentous actin in active flux, as described for other cells (Carlier, 1998). In many retinal neuroepithelial cells, however, fluorescent actin accumulations appear and stay throughout the whole imaging period (240 s). Most, but not all, of these accumulations can be observed basal to the nucleus (Figure 6B and Movie S14).

MyosinII is a common actin-associated force-generating motor protein in nonmuscle cells. Therefore, it is a good candidate to investigate whether actomyosin networks play a role in IKNM. To probe where MyosinII is active in neuroepithelia cells, we performed antibody staining against Phospho-Myosin Light Chain 2, which stains only the active form of MyosinII. Colabeling with 4′, 6-diamidino-2-phenylindole (DAPI) revealed a concentration of active MyosinII immunoreactivity just basal to the nucleus in some cells (Figure 6C, arrows). As these were fixed embryos, we were unable to draw any conclusions about whether such nuclei were moving apically or basally. To address this, we used MRLC2^T18DS19D^-GFP, a tagged constitutively activated regulatory light chain, (Iwasaki et al., 2001). This construct localizes as bright dots just basal to nuclei that are moving apically (Figure 6D and Movie S15). This localization of MRLC2^T18DS19D^-GFP, but not nonactivatable MRLC2^T18AS19A^-GFP (Iwasaki et al., 2001), overlaps with RFP-Utr-CH (Figures S3B and S3C). Overall, the localization of actin and activated MyosinII suggests that this cytoskeletal component may have a role in IKNM.

### Inhibition of MyosinII Inhibits IKNM

**Figure 7:**
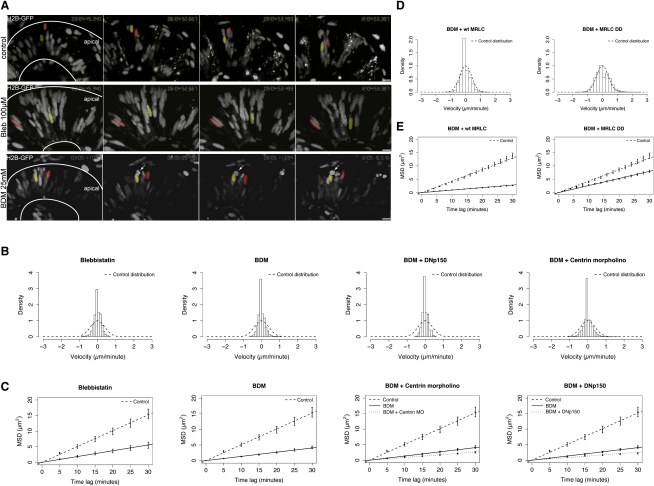
IKNM Movements Are Inhibited when MyosinII Activity Is Blocked, but the Effect Can be Rescued by MRLC2^T18DS19D^ (A) Images from Movies S13–S15 at 3 hr intervals. Pseudocolored nuclei in WT (upper panel) travel further than nuclei treated with 100 μM Blebbistatin or 25 mM BDM (lower panels). Arrows mark a mitotic but not separated nucleus in BDM treatment. (B) Stochastic motion velocity distributions for Blebbistatin- and BDM-treated embryos, and centrin morphants and DNp150-expressing cells treated with BDM. (C) Stochastic motion MSD profiles (error bars show 95% confidence intervals) with linear fit for Blebbistatin- and BDM-treated embryos, and centrin morphant and DNp150-expressing cells treated with BDM. (D) Stochastic motion velocity distributions for BDM-treated embryos injected with MRLC2^T18DS19D^-GFP or wild-type MRLC2-GFP, respectively. (E) Stochastic motion MSD profiles (error bars show 95% confidence intervals) with linear fit for BDM-treated embryos injected with MRLC2^T18DS19D^-GFP or MRLC2-GFP, respectively. Only the activated MRLC partially rescues the effect of BDM treatment. Scale bars represent 10 μm.

If actomyosin forces drive IKNM, then inhibition of MyosinII should significantly impact nuclear movements. To test this, we used two pharmacological inhibitors of MyosinII activity, Blebbistatin and BDM. Addition of either blocked cytokinesis, which is known to depend on actomyosin activity (Figure 7A, arrow). After MyosinII inhibition, we first examined actin dynamics by photoactivating a spot of PA-GFP-Utr-CH in retinal neuroepithelial cells. We found that the majority of actin rapidly disperses as in control cells, but that the stable actin accumulations basal to the nucleus do not persist in BDM-treated embryos as they do in control cells (Figure S4).

In MyosinII-inhibited cells, both instantaneous nuclear velocity and MSD are greatly reduced compared to control cells (Figures 7B and 7C and Movies S16–S18). In addition, not a single event of rapid apical migration could be found in all the BDM and Blebbistatin movies. Nuclei that stalled in cytokinesis were found at the apical surface, having migrated apically before the drugs took full effect. These data thus strongly implicate actomyosin forces as major drivers of IKNM. If this striking reduction in IKNM dynamics is due to the specific inhibition MyosinII activity, it should be rescued by providing BDM insensitive MyosinII. We therefore coinjected the constitutively activated Myosin Light Chain MRLC2^T18DS19D^-GFP described above with H2B-RFP into a subset of cells and treated these embryos with BDM. BDM inhibits the ATPase activity of MyosinII (Cramer and Mitchison, 1995), whereas phosphorylated MRLC enhances the actin-activated ATPase activity of MyosinII, thus counteracting to some extent the effects of BDM (Bresnick et al., 1995). As a control, we did the same experiment using the wild-type version of MRLC, MRLC2-GFP. Trajectories of nuclei in both conditions were measured. As seen in Figures 7D and 7E and Movie S19, the activated version of MRLC partially rescues the BDM effects, whereas the wild-type version does not, consistent with the IKNM-inhibiting effects of BDM being due to specific inhibition of MyosinII.

### A Minor Role for MTs in IKNM Is Revealed in MyosinII-Inhibited Cells

In Blebbistatin- and BDM-treated embryos, although the MSDs were greatly reduced, there were still some stochastic movements remaining. We therefore asked whether these remaining movements were dependent on MTs or their associated motors. To test this, we added BDM to either centrin morphants or to embryos expressing DNp150. As in cells in which MyosinII alone was inhibited, we never found rapid persistent nuclear migration in cells deficient for both MyosinII activity and MT-motor coupling. However, nuclear velocities and MSDs were reduced slightly below values of cells treated with BDM alone (Figures 7B and 7C). Interestingly, this further reduction approximates the reduction we saw when we compared the DNp150 or centrin morphants alone to the control situation. Thus, these experiments substantiate that MTs and their associated motors play a role in IKNM dynamics, but clearly the bulk of IKNM dynamics are dependent on actomyosin.

## Discussion

We imaged nuclear movements associated with IKNM at high temporal and spatial resolution in the zebrafish retinal neuroepithelium. Statistics describing fundamental properties of nuclear trajectories argue that nuclear movements are stochastic rather than directed throughout more than 90% of the cell cycle. Only in G2, before mitosis, do we find brief periods of directed apical nuclear motion. Our results thus challenge the smoothly running “elevator” model of nuclear movements during IKNM. We instead propose a new analogy: in our opinion, IKNM is reminiscent of movements of people at a crowded party held in a room with a bar at one end. When people get thirsty, they go to the bar to get a drink. Then they move or are pushed away to make room for others at the bar. Between drinks the partygoers jostle around the room, but when they get thirsty again, they return to the bar in a fast and directed manner.

Immunolabeling, electron microscopy, and live imaging studies reveal oriented MTs that extend from the apically located centrosome along the apicobasal axis of neuroepithelial cells, passing within molecular distances of the nucleus. It has been suggested that an oriented population of MTs, in concert with plus or minus end-directed motor protein complexes, could provide a mechanism for transport of the nucleus (Xie et al., 2007). However, we find that major features of IKNM still occur in the complete absence of MTs. In contrast, we found that after the application of drugs that inhibit MyosinII, the stochastic nuclear movements are greatly reduced and rapid directed events are eliminated. These results indicate that both the nondirected movements that dominate interphase and the persistent apical movements that occur in G2 are actomyosin dependent. The fact that we can partially rescue the effects of BDM by overexpressing constitutively active MRLC corroborates this conclusion.

The observations that nuclear movements are slightly reduced when stable MTs are removed either in otherwise untreated cells or in the presence of BDM supports the idea that MTs play a role in IKNM. It has also previously been shown that nuclei of dynein/dynactin-compromised neuroepithelial cells migrate to more basal positions than nuclei from control cells (Del Bene et al., 2008). We confirmed this finding using DNp150 under a heat shock promoter, and saw in DNp150-expressing cells occasional very rapid basally directed movements, which could explain the basal misposition phenotype. Dynein/dynactin might normally counteract these rapid and direct movements to basal positions, possibly by linking the nucleus to the MT cage. Dynein/dynactin does not, however, provide the main motor for rapid direct apical migration preceding mitosis. In fact, in DNp150-expressing cells, these apical movements tended to be more rapid than those in control cells. Nuclei in DNp150-expressing cells “start” from more basal positions and must therefore translocate further than control nuclei. Our results showing faster apical migrations in DNp150-expressing cells contradict those of Del Bene et al. (2008) indicating that apical nuclear migration is slower in *mok* mutants. The different results are most probably explained the fact that Del Bene et al. only measured nuclear movements at high temporal resolution for 1 hr, and thus could have easily missed the rapid premitotic apical migrations that only happen once per cell cycle.

Our results suggest that once the signal to move apical and undergo mitosis is received, nuclei, no matter where they are situated along the apicobasal axis, must travel all the way to the apical pole where the centrosomes, which are necessary to make the spindle poles, are stationed. Thus, nuclei that start more basally, such as in the DNp150 cells, must travel faster. Nuclei that start more apically, such as in Colcemide-treated cells, may thus travel more slowly during this period.

Though we have identified actomyosin as the prime mover of IKNM, many questions remain. For example, we do not understand to what extent the stochastic movements are intrinsically driven by actomyosin forces within individual cells. It is possible that most stochastic movements derive from the nudges of neighboring cells. Stochastic movements might therefore, like a form of Brownian motion, be an emergent property of the epithelium rather than an intrinsically stochastic process generated within each cell. A closer look at actomyosin forces in individual cells might help to answer these questions.

What triggers actomyosin activity that leads to rapid apical migration in G2 is another open question. Almost all actomyosin-triggered events are calcium dependent, so it is interesting to note that individual precursor cells display calcium fluctuations (Owens and Kriegstein, 1998) and it has been shown that the speed of nuclear movements is reduced when calcium transients are inhibited (Pearson et al., 2005). It will thus be exciting to test whether calcium transients cause IKNM movements. It may also be interesting to analyze whether kinesin motors, like dynein/dynactin, have augmenting functions in IKNM.

## Experimental Procedures

### Animals

Zebrafish were maintained and bred at 26.5°C, and embryos were raised at 28.5°C and staged as previously described (Kimmel et al., 1995) in hpf. Embryos were treated with 0.003% phenylthiourea (Sigma) from 11–24 hpf to delay pigmentation. All animal work was approved by Local Ethical Review Committee at the University of Cambridge and performed according to the protocols of project license PPL 80/2198 approved by the UK Home Office.

### Constructs and Antibodies

The EMTB-GFP (Bulinski et al., 1999), EB3-GFP (Stepanova et al., 2003) and γ-tubulin-YFP subcloned into the pCS2+ vector were kind gifts from Virginie Lecaudey in Darren Gilmour's lab. The H2B-RFP construct has been published and was subcloned into the pCS2+ vector. For the hsp70-PEST-DNp150-GFP construct, −1.5 kb of the *hsp70* promoter from zebrafish was used to replace the CMV promoter of the pd2EGFP-N1 vector (ClonTech). The complementary DNA (cDN)A for human dominant-negative, C-terminal truncated dynactin, corresponding to amino acids 1–811 (Vaughan et al., 2001), was then placed 3′ to the pd2EGFP (destabilized GFP) coding sequence to generate an N-terminally tagged fusion protein, pd2EGFP-dnct1^1–811^. Utr-CH-GFP, Utr-CH-RFP, and PaGFP-Utr-CH were a kind gift from the von Dassow lab and have been published (Burkel et al., 2007). MRLC2^T18DS19D^-GFP and MRLC2^T18AS19A^-GFP were a kind gift from the Hosoya Lab (Iwasaki et al., 2001) and have been subcloned into the pCS2+ vector with NotI, blunted with Klenow and EcoRI (Stu1 and EcoR1 for the pCS2+ vector).

Anti-α- and acetylated tubulin antibodies (Sigma, Abcam, UK) were used in a 1:250 dilution. Anti-Phospho-Myosin Light Chain II antibody (Cell Signaling, UK) was used in a 1:50 dilution. All secondary antibodies were used in a 1:1000 dilution. Secondary antibodies used were goat anti-mouse or anti-rabbit IgG conjugated to Cy3 (Chemicon, Temecula, CA) or conjugated to Alexa 488, 594 fluorophores (Molecular Probes, Eugene, OR). Nuclei were counterstained with DAPI.

### DNA, RNA and MO Injections and Whole-Mount Staining

In general, DNA and MO injections were performed at the one cell stage. Injections of fluorescently labeled RNA were carried out at the 8–64 cell stage to ensure a mosaic expression pattern. Centrin2-MOs were injected at a concentration of 2–3 ng. The sequence start morpholino (ATG underlined) was TTTCCTGAAGCCGGACGCCATTTTG, and the sequence splice morpholino (exon 3, intron 3) was TGTTTGGTTTGCTCACCATTTTCTG. Whole-mount staining of zebrafish embryos was carried out by fixation of 24 hpf embryos in 4% paraforemaldehyde at 4°C over night. Three times 30 min in PBS 0.2% Triton followed by a 15 min digest with Trypsin EDTA on ice was used for permeabilization. The following washing steps were done in PBS 0.2% Triton. Blocking was done in 10% goat serum, 1% BSA, and 0.2% Triton. ntibodies were added in blocking solution. First antibodies were added for 72 hr at 4°C, and second antibodies were added for 48 hr.

### Heat Shock Experiments

Microinjection of the hsp70-PEST-DNp150-GFP construct was performed into the cytoplasm of single-cell-stage embryos. After injections, embryos were incubated at 28.5°C until 24 hpf. Embryos were then heat shocked for 1 hr in a water bath at 39°C.

### Drug Treatments

Embryos were mounted in 1% agarose and covered in embryo medium. Colcemide (Sigma) was prepared as a 0.2 mM stock in DMSO and used at 20 μM, Blebbistatin (Sigma) was prepared as a 1 mM stock in DMSO and used at 100 μM, and BDM (Calbiochem) was prepared as a 500 mM stock in DMSO and used at 25 mM. Control embryos were treated in the same manner and exposed to a similar concentration of carrier solvent (DMSO) without their being an effect on nuclear migration. Colcemide and BDM were added and left in the embryo medium. Heartbeat was still observed during the analysis time of the movies. Blebbistatin was added to the medium and then washed out. Ten hours after wash out, IKNM recovered to normal rates (data not shown), arguing that the Blebbistatin effect was reversible.

### Ultrastructure Analysis of MTs and Nuclei

Embryos (24 hpf) were cut and fixed by immersion in 2% glutaraldehyde containing 2 mmol/l Ca Cl2 in 0.1 M PIPES buffer at pH 7.4. H2O2 (100 μl of 33%) was added to each 10 ml aliquot immediately before use. They were fixed in suspension for 2 hr at 4°C. They were rinsed twice in buffer (0.1 M PIPES) and transferred to 1.5 ml glass tubes. Then they were postfixed in 1% osmium ferricyanide for 1 hr, rinsed three times in DIW, and bulk stained in 2% uranyl acetate for 1 hr. They were rinsed in DIW and dehydrated in an ascending series of ethanol solutions to 100% ethanol, rinsed twice in acetonitrile, and embedded in quetol epoxy resin. Fifty nanometre sections were cut on a Leica Ultracut UCT, stained with saturated uranyl acetate in 50% ethanol and lead citrate, and viewed in an FEI Philips CM100 operated at 80 kv.

### In Vivo Imaging

Embryos were prepared for imaging as described (Poggi et al., 2005). Fluorescence in the specimens was imaged with a confocal laser-scanning microscope (model TCS-NT; Leica) with a 63× NA 1.2 water immersion objective (Leica). Spinning disk movies used a Perkin Elmer Spinning disk microscopes UltraVIEW VoX Zeiss AxioVert 200M Inverted microscope and a 40×/1.3 NA U Plan Semi Apo objective or an UltraVIEW ERS, Olympus IX81 Inverted microscope, and a 40×/1.3 NA U Plan Semi Apo objective. Optical sections 1 μm apart were taken through a volume of the retina up to 30 μm in depth. Time points were between 10 s and 5 min apart. The four-dimensional data thus obtained was processed and analyzed with the Volocity software (Improvision, Coventry, UK). Quantifications described in the text were obtained with ImageJ (National Institutes of Health) software. Photoactivation of the PaGFP-Utr-CH construct was carried out on the UltraVIEW VoX Zeiss AxioVert 200M Inverted system using a 405 laser pulse for 100–200 bleaching cycles.

### Statistical Analysis of Nuclear Motion

Apicobasal nuclear position trajectories were generated where nuclear position at each time point (*p_i_*) is defined as the straight line distance between the point of cell contact with the basal lamina, and the basal extent of the nucleus. Positions were measured at time intervals of 2, or 5 min, for periods of 1 to 13 hr. In drug-treated embryos, we only consider nuclear movement >120 min after drug addition. Statistical analyses were performed using custom routines in R (R Development Core Team, 2009).

#### Instantaneous Velocity Distributions

We transform our time series of one-dimensional nuclear positions into series of nuclear displacements by taking the first difference and dividing by the time interval to define an instantaneous velocity series (p, position; *t*, time; and *i*, time point):

$$v_{i}=\frac{\left(p_{i+1}-p_{i}\right)}{\left(t_{i+1}-t_{i}\right)}\text{.}$$

Positive (negative) values represent apical (basal) movements. Instantaneous velocity distributions were plotted as histograms. Nonparametric density estimates were generated using a Gaussian smoothing kernel.

#### Identification of Distinct Directed and Stochastic Motion Types in Nuclear Trajectories

We employ a moving analysis window strategy inspired by that of Huet et al. (2006) to objectively detect transient periods of rapid persistent motion that would have a negligible probability (<0.01) of arising through random motion. This allows us to isolate stochastic and directed movement periods and examine their occurrence/properties in different treatment groups. See the Supplemental Experimental Procedures for details.

#### Mean Squared Displacement Calculation

We use the MSD as a function of elapsed time as a simple statistical measure of the average distance a nucleus travels over time. Trajectories representing each treatment group were pooled to calculate MSD profiles. See the Supplemental Experimental Procedures for details.

#### Proportional Nuclear Position 30 min before Mitosis

We normalize by the cell length to define proportional nuclear position. We examine the distribution of proportional nuclear positions 30/32 min before formation of the metaphase plate, the approximate time at which nuclei transition from stochastic to persistent apical movement.

#### Velocity of Persistent Apical Movement before Mitosis

The velocity of persistent apical movement preceding mitosis was estimated as the slope of a linear regression line fitted to the nuclear trajectory between −30/32 and 0 min relative to formation of the metaphase plate.

## Supplemental Data

Document S1. Supplemental Experimental Procedures, Four Figures, and Two Tables

Movie S1. Representative In Vivo Confocal Time-Lapse Movie of Interkinetic Nuclear Migration in a Control Retinal Neuroepithelium Nuclei are labeled by H2B-RFP. Two representative nuclei are pseudocolored. The movie begins at 32 hpf and plays at seven frames per second at 2 min intervals, covering 5 hr and 30 min of developmental time.

Movie S2. In Vivo Confocal Time-Lapse Movie Showing EMTB-GFP in a Control Retinal Neuroepithelium The embryo is 28 hpf and the movie plays at seven frames per second, covering 14 min 47 s at 40 s intervals. No obvious intensity changes can be observed.

Movie S3. In Vivo Confocal Time-Lapse Movie Showing EB3-GFP in a Control Retinal Neuroepithelium The embryo is 28 hpf, and the movie plays at 15 frames per second, covering 33 min at 20 s intervals. Almost all EB3 is going in apical basal direction. Bright dots at the apical side label the centrosome.

Movie S4. In Vivo Confocal Time-Lapse Movie Showing the Centrosome Labeled by Centrin2-GFP and the Nucleus Labeled by H2B-RFP in a Control Retinal Neuroepithelium The embryo is 28 hpf at the start of the movie. The movie plays at nine frames per second, covering 3 hr 48 min of development at 4 min intervals. Centrin2 is localized apically the whole course of the movie, and only leaves this site during mitosis. Nuclei migrate apically and basally.

Movie S5. In Vivo Confocal Time-Lapse Movie Showing EB3-GFP in a Centrin2 Splice Morphant Neuroepithelium The embryo is 28 hpf, and the movie plays at 15 frames per second, covering 20 min 35 s at 12 s intervals. Almost all EB3 is going in apical basal direction. Bright dots at the apical side label the centrosome.

Movie S6. In Vivo Confocal Time-Lapse Movie Showing the Centrosome Labeled by γ-Tubulin YFP and the Nucleus Labeled by H2B-RFP in the Centrin2 Splice Morphant Retinal Neuroepithelium The embryo is 28 hpf at the start of the movie. The movie plays at seven frames per second, covering 38 min at 2 min intervals. γ-tubulin YFP is localized apically the whole course of the movie, and only leaves this site during mitosis. Nuclei migrate apically and basally.

Movie S7. Representative In Vivo Confocal Time-Lapse Movie of Interkinetic Nuclear Migration in Centrin2 Translational Morphant Retinal Neuroepithelium Nuclei are labeled by H2B-RFP. Two representative nuclei are pseudocolored. The movie begins at 32 hpf and plays at seven frames per second at 2 min intervals, covering 5 hr of developmental time.

Movie S8. In Vivo Confocal Time-Lapse Movie Showing Fast Apical Migration in Centrin 2 Translational Morphant Retinal Neuroepithelium Nuclei are labeled by H2B-RFP. The apically migrating nucleus is pseudocolored. The movie plays at 2 min intervals and covers 100 min.

Movie S9. In Vivo Confocal Time-Lapse Movie of Interkinetic Nuclear Migration in a Mosaic Centrin2 Translational Morphant Retinal Neuroepithelium Nuclei of cells that have centrin2 morpholino are labeled by H2B-RFP; nuclei of cells that have no morpholino are labeled by H2B-YFP. The movie begins at 32 hpf and plays at seven frames per second at 2 min intervals, covering 6 hr of developmental time.

Movie S10. In Vivo Confocal Time-Lapse Movie Showing EB3-GFP in the Neuroepithelium of an Embryo Treated with 20 mM Colcemide The embryo is 28 hpf and the movie plays at 12 frames per second at 2 min intervals, covering 5 hr 14min of development. EB3 starts to disappear 120 min after addition and cannot be seen moving into an apicobasal direction 180 min and more after drug addition. The first arrows mark a proper spindle formation and division between 68 min and 94 min. The later arrows mark a cell trying to form a spindle but failing starting at 160 min and being stuck in metaphase until the end of the movie.

Movie S11. Representative In Vivo Confocal Time-Lapse Movie of Interkinetic Nuclear Migration in a with 20 mM Colcemide-Treated Retinal Neuroepithelium Nuclei are labeled by H2B-RFP. The movie begins at 32 hpf and plays at seven frames per second at 2 min intervals, covering 3 hr and 28 min of developmental time. Colcemide was added at the beginning of the movie and not removed. Movie starts at 3 hr 36 min after Colcemide depolymerized MTs.

Movie S12. In Vivo Confocal Time-Lapse Movie Showing Fast Apical Migration in a Retinal Neuroepithelium Treated with 20 mM Colcemide Nuclei are labeled by H2B-RFP. The apically migrating nucleus is pseudocolored. The movie plays at 2 min intervals covers 60 min.

Movie S13. In Vivo Confocal Time-Lapse Movie Showing GFP-UtrCH in a Control Retinal Neuroepithelium The embryos is 32 hpf and the movie plays at 12 frames per second, covering 5 hr of development at 2 min intervals. GFP-UtrCH marks the plasma membrane of the whole cell during all of the cell cycle, except when cells go into mitosis. During this time GFP-UtrCH disappears from the basal process and only gets back after cell division.

Movie S14. In Vivo Confocal Time-Lapse Movie Showing PA-GFP-UtrCH in a Control Retinal Neuroepithelium The embryos is at 28 hpf and the movie plays at ten frames per second, covering 6 min at 6 s intervals. Nuclei are labeled by H2B-RFP. PA-GFP-UtrCH is activated by a 405 mn laser pulse at 36 s. Most actin moves down the basal process but some also distributed to the apical side of the cell. Stable actin accumulations can be observed.

Movie S15. In Vivo Confocal Time-Lapse Movie Showing MRLC2T18DS19D-GFP in a Control Retinal Neuroepithelium Nuclei are labeled by H2B RFP. The embryos is 28 hpf and the movie plays at seven frames per second, covering 90 min at 2 min intervals. The pink dot labels the nucleus that migrates apically. Arrows label following activated Myosin marked by MRLC2^T18DS19D^-GFP.

Movie S16. Representative In Vivo Confocal Time-Lapse Movie of Interkinetic Nuclear Migration in a Control Retinal Neuroepithelium Nuclei are labeled by H2B-RFP. Individual nuclei are pseudocolored red or yellow. The movie begins at 32 hpf and plays at five frames per second at 30 min intervals, covering 8 hr of developmental time.

Movie S17. Representative In Vivo Confocal Time-Lapse Movie of Interkinetic Nuclear Migration in a Retinal Neuroepithelium Treated with 100 mM Blebbistatin Blebbistatin was added at the beginning of the movie and removed after 30 min. Nuclei are labeled by H2B-RFP. Individual nuclei are pseudocolored red or yellow. The movie begins at 32 hpf and plays at five frames per second at 30 min intervals, covering 8 hr of developmental time.

Movie S18. Representative In Vivo Confocal Time-Lapse Movie of Interkinetic Nuclear Migration in a Retinal Neuroepithelium Treated with 25 mM BDM BDM was added at the beginning of the movie and not removed. Nuclei are labeled by H2B-RFP. Individual nuclei are pseudocolored red or yellow. The movie begins at 32 hpf and plays at five frames per second at 30 min intervals, covering 8 hr of developmental time.

Movie S19. Representative In Vivo Confocal Time-Lapse Movie of Interkinetic Nuclear Migration in a Retinal Neuroepithelium Treated with 25 mM BDM BDM was added at the beginning of the movies and not removed. Nuclei are labeled by H2B-RFP. The upper panel shows nuclei in an embryo that also expresses MRLC2-GFP in cells with labeled nuclei. The lower panel shows an embryo that also expresses MRLC2^T18DS19D^-GFP in cells with labeled nuclei. The movie begins at 32 hpf and plays at seven frames per second at 2 min intervals, covering 7 hr of developmental time. Note that nuclei in the MRLC2^T18DS19D^-GFP epithelium keep moving during the whole course of the movie while nuclei in the MRLC2-GFP epithelium almost stop completely.
