# Supplementary material for: Actomyosin Is the Main Driver of Interkinetic Nuclear Migration in the Retina
Source: Cell. 2009 Sep 18;138(6-10):1195–208. doi: 10.1016/j.cell.2009.06.032 (PMC2791877; doi:10.1016/j.cell.2009.06.032)
Supplement: Document S1. Supplemental Experimental Procedures, Four Figures, and Two Tables [file mmc1.pdf]

Cell, Volume *138*

Supplemental Data

Actomyosin Is the Main Driver of Interkinetic Nuclear Migration in the Retina

Caren Norden, Stephen Young, Brian A. Link, and William A. Harris

Supplemental Experimental Procedures

Transient motion analysis

A proportion of nuclei were seen to engage in transient periods of directed and rapid movement. These transient periods occur in a background of stochastic motion. We employ a quantitative method to identify these transient periods within individual trajectories (Huet et al., 2006). Linear filtering using a moving average isolates the trend in a time series (Diggle, 1994). The trend in nuclear position was measured using an equally weighted moving average over a window of t ± 5/6 minutes. A moving analysis window (±14/15 minutes, matched to the approximate duration of transient movement periods), scans the full length of the trajectory and assigns a score to the central time point of each subtrajectory, where the score is the slope of the trend over the subtrajectory (evaluated by linear regression).

Because random walks can temporarily mimic directed movement, the method used to identify non-random movement periods must incorporate a threshold to exclude false positives (Saxton, 1994). The threshold is selected through analysis of simulated random walk controls. One thousand one-dimensional random walks of one hundred steps were simulated using steps randomly sampled from a gaussian distribution fitted by maximum likelihood to the nuclear displacement distribution from control embryos. The transient motion analysis algorithm was applied to random walk controls, and the threshold was set so that a random walk subtrajectory would have negligible probability (<0.01) of being identified as non-random motion.

Mean squared displacement calculation and model fitting

Where δt is the time interval between consecutive measurements of nuclear position, the mean squared displacement for time lag $\Delta t = n\ \delta t$ (where n is an integer), may be calculated for an individual trajectory by averaging over all pairs of time points Δt apart (Qian et al., 1991; Saxton, 1997)

$$MSD(\Delta t) = \frac{1}{N-n}\sum_{i=1}^{N-n}\left[p((i+n)\delta t) - p(i\delta t)\right]^2$$

where N is the total number of time points in the trajectory. The larger the value of Δt, the fewer displacements are averaged, and consequently the reliability of the MSD measure decreases. To maximize the reliability of the MSD measure within treatment groups, periods of stochastic movement from each treatment group were concatenated, and mean squared displacements were calculated along these composite trajectories (Jaqaman et al., 2006).

We fit a power law model ($MSD(\Delta t) = 2D\Delta t^{\alpha}$) to mean square displacement profiles for stochastic movement periods, estimating D and α by linear regression of ln(MSD) versus ln(time). In log form, $\ln(MSD(\Delta t)) = \ln(2D) + \alpha \ln(\Delta t)$, the intercept and slope correspond to ln(2D) and α respectively. Values of alpha close to one are consistent with a stochastic diffusive behaviour. In order to quantitatively compare diffusive model fits, we fit the same data but constrain alpha to take the value 1 and re-evaluate D as the average of $\frac{MSD(\Delta t)}{2\Delta t}$. Parabolic fits corresponding to a one-dimensional diffusion with flow model ($MSD(\Delta t) = 2D\Delta t + v^2 \Delta t^2$) were fit by quadratic regression of MSD versus time with intercept 0.

Supplemental References

Diggle, P. J. (1994). Analysis of Longitudinal Data. Oxford University Press, USA.

Huet, S., Karatekin, E., Tran, V. S., Fanget, I., Cribier, S., and Henry, J. P. (2006). Analysis of transient behavior in complex trajectories: application to secretory vesicle dynamics. Biophys J *91*, 3542-3559.

Jaqaman, K., Dorn, J. F., Jelson, G. S., Tytell, J. D., Sorger, P. K., and Danuser, G. (2006). Comparative autoregressive moving average analysis of kinetochore microtubule dynamics in yeast. Biophys J *91*, 2312-2325.

Qian, H., Sheetz, M. P., and Elson, E. L. (1991). Single particle tracking. Analysis of diffusion and flow in two-dimensional systems. Biophys J *60*, 910-921.

Saxton, M. J. (1994). Single-particle tracking: models of directed transport. Biophys J *67*, 2110-2119.

Saxton, M. J. (1997). Single-particle tracking: the distribution of diffusion coefficients. Biophys J *72*, 1744-1753.

Figure S1.

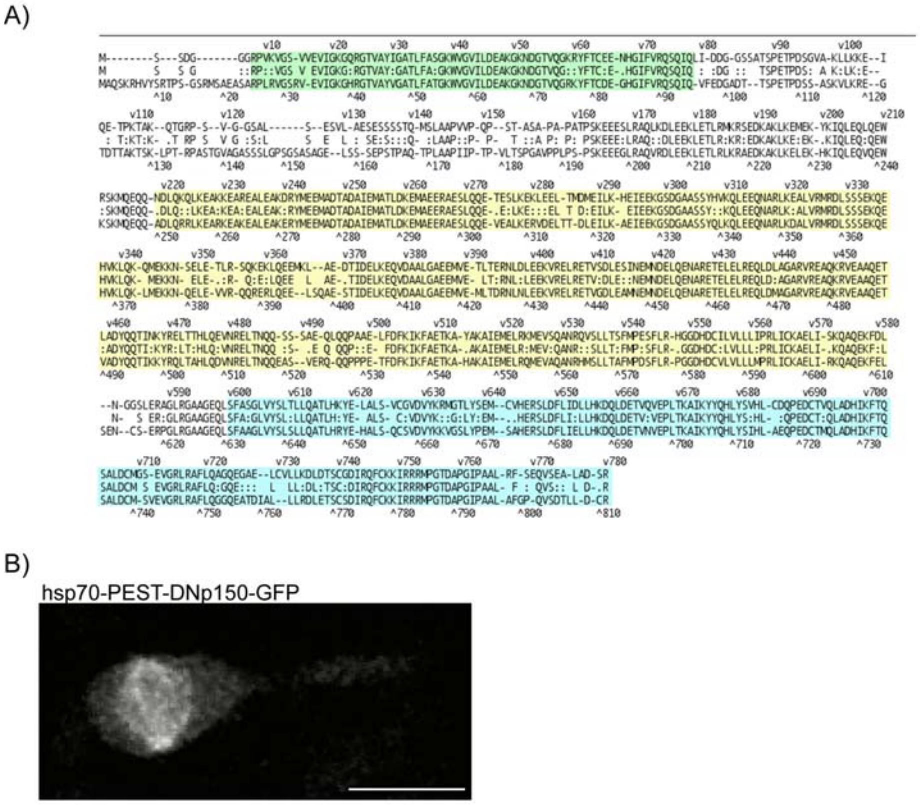

A) Alignment of Dnct1 from zebrafish (top sequence) versus human (bottom sequence) for the region of the dominant negative protein. Alignments were generated in MegAlign (DNAstar, V6) using the Lipman-Pearson protein algorhithm with 0 gap penalty. Overall sequence identity between the proteins is 65%. Within specific domains, however, identity is higher: Microtubule binding domain/Cap-Gly region, 86% identity (green); dynein motor binding domain, 84% identity (yellow); dynein intermediate chain binding domain, 80% identity (blue).

B) The dominant negative p150-GFP labels spindle structures at mitosis.

Figure S2.

A)

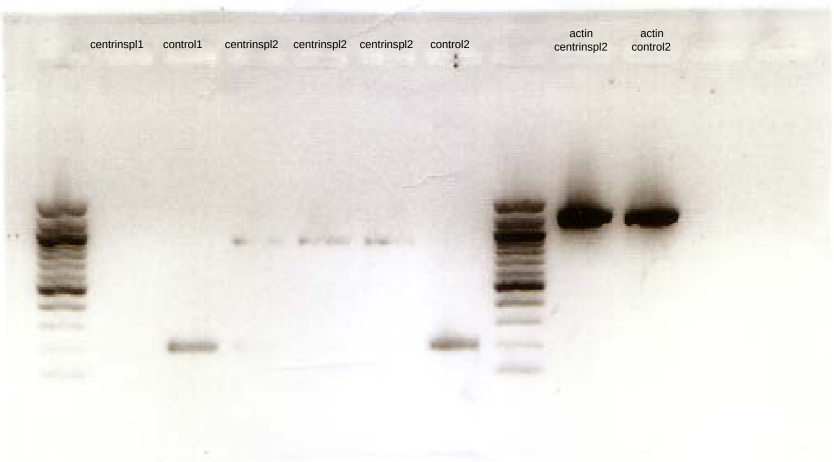

B)

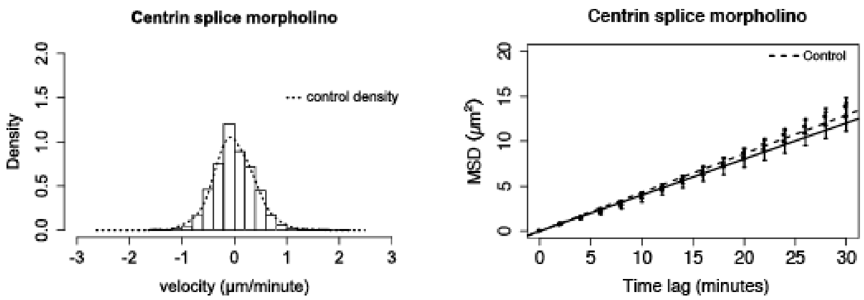

C)

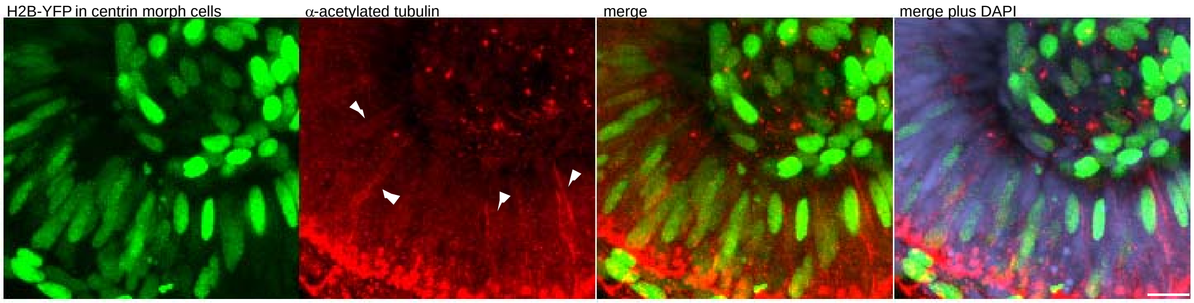

A) RNA was extracted from embryos using a Quiagen RNeasy Minikit and transcribed into DNA using the Quiagen One Step RT-PCR-kit.
Centrin DNA primers used:
F CGCTGATATTGATAAAGAAGGATCGGGC
R CATCCGTCAGGTTCTCTCCGAGC
Control embryos give a 200 bp band whereas incorporation of the intron in the splicing morphant gives a 900 bp band.As no 200bp band can be observed any more in the splicing morphant we conclude that the knockdown of centrin2 is nearly complete.
Beta actin DNA primers, that result in a 1200 bp product are used as control:
F GATGCCCCTCGTGCTGTTTTC
R ACCTCCCTTTCCAGTTTCCGC

B) Stochastic motion velocity distributions for centrin splice morphants and stochastic motion MSD profiles with linear fit for centrin splice morphants

C) Embryos were co-injected with H2B-YFP-RNA and 2.5ng Centrin2 translational morpholino at the 64 cell stage. Only cells expressing H2B-YFP are centrin morphants. Antibody staining for stable (acetylated) tubulin (red) was carried out to observe if cells that do not carry the morpholino still form stable microtubules that span the whole length of the cell. DAPI (blue) counterstaining shows cells not expressing H2B-YFP.

Figure S3.

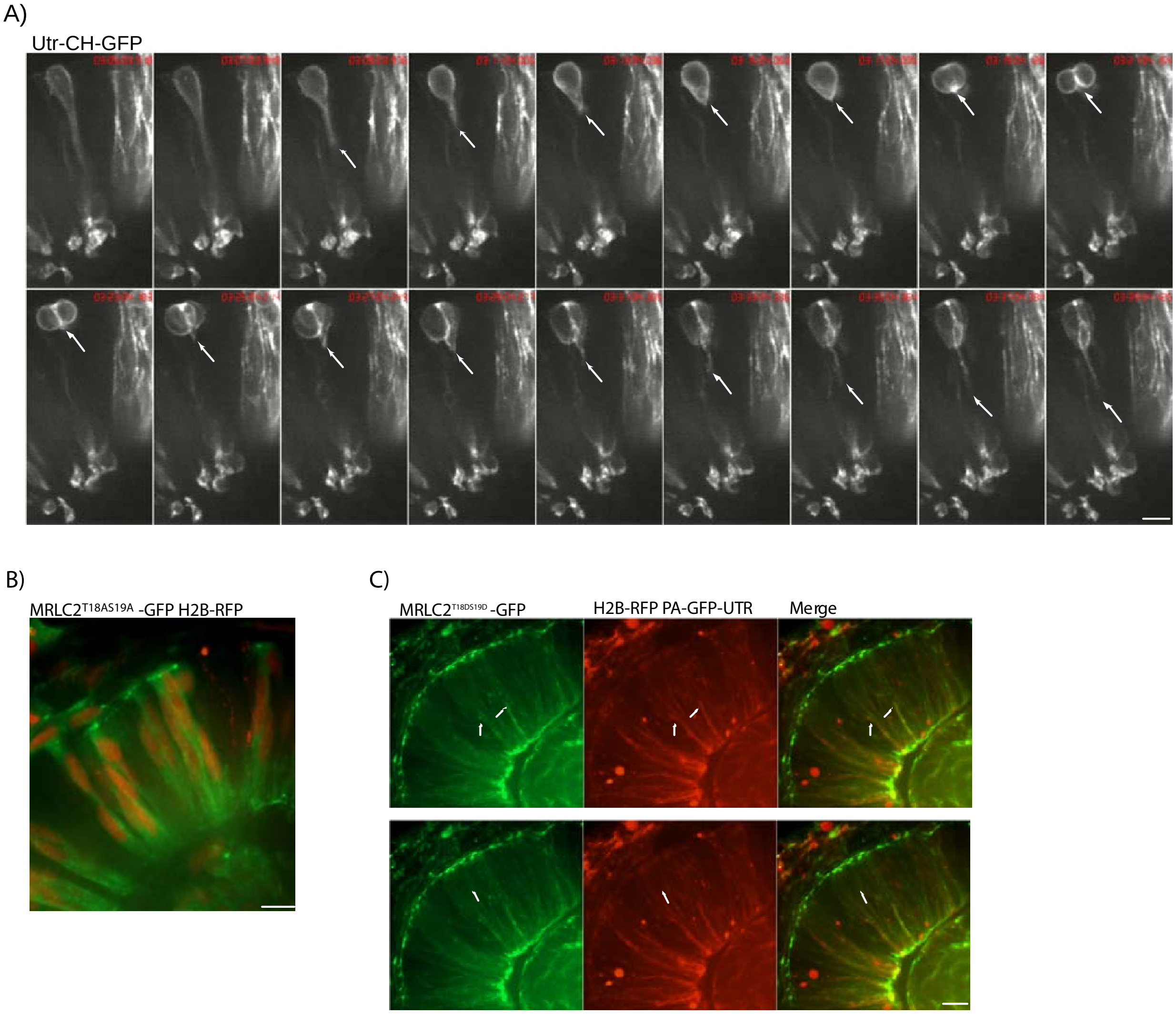

A) Images from Movie 10. GFP-Utr-CH disappears from the basal process of cells just before mitosis and cytokinesis (arrows moving up). It quickly reappears after cell division (arrows moving down).

B) Non-activatable Myosin is labelled by MRLC2T18AS19A-GFP. Nuclei are labelled by H2B-RFP.
No dot like accumulations basal of nuclei can be observed.

C) Co-labelling of MRLC*2T18DS19D*-GFP for constitutively activated Myosin and RFP-Utr-CH for filamentous actin shows that localizations overlap (merge). Arrows show basal accumulation of activated myosin with actin accumulations.

Figure S4.

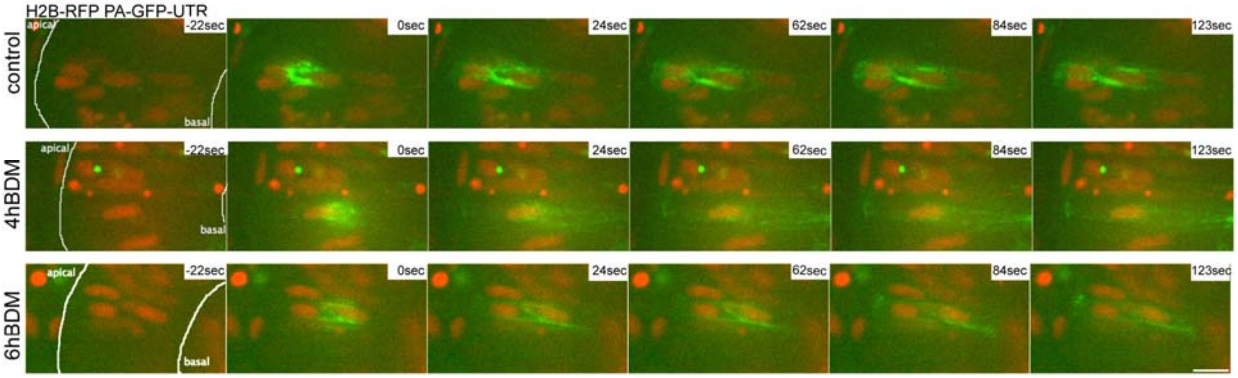

Photoactivation experiments of PaGFP-Utr-CH in control epithelia; epithelia treated with 25mM BDM for 4h and epithelia treated with 25mM BDM for 6h are compared. The GFP marker gets more diffuse the longer BDM is added and no stable accumulations of filamentous actin are observed any more.
H2B-RFP is used to label nuclei.

Table S1.

Number of embryos and nuclei analysed						
	Total		2-minute sampling		5-minute sampling	
**Treatment**	**Embryos**	**Nuclei**	**Embryos**	**Nuclei**	**Embryos**	**Nuclei**
Control	5	32	3	15	2	17
BDM	4	20	-	-	3	15
Blebbistatin	3	18	-	-	3	18
Colcemide	5	23	5	23	-	-
Centrin trans mo	3	16	1	6	2	10
Centrin splice mo	4	21	1	5	3	16
DNp150	10	35	-	-	10	35
BDM+centrin mo	6	30	3	15	3	15
BDM+DNp150	3	14	-	-	3	14
BDM+wt MRLC	4	20	4	20	-	-
BDM+MRLC DD	4	22	4	22	-	-
Mosaic control	3	15	3	15	-	-
Mosaic centrin mo	3	15	3	15	-	-

Table S2.

Average individual nuclear trajectory length and length of composite trajectory used for MSD calculation				
	2-minute sampling		5-minute sampling	
**Treatment**	**Median length (mins)**	**Composite length (mins)**	**Median length (mins)**	**Composite length (mins)**
Control	232	3848	410	5935
BDM	-	-	460	6815
Blebbistatin	-	-	235	3810
Colcemide	174	3898	-	-
Centrin trans mo	300	1772	265	2725
Centrin splice mo	272	1298	245	4400
DNp150	-	-	230	9300
BDM+centrin mo	362	5184	400	6050
BDM+DNp150	-	-	362.5	4555
BDM+wt MRLC	318	6048	-	-
BDM+MRLC DD	305	5752	-	-
Mosaic control	298	4956	-	-
Mosaic centrin mo	326	4840	-	-
